# Perfusate Flow Rate: An Overlooked Factor in Hyperthermic Intraperitoneal Chemotherapy (HIPEC)?

**DOI:** 10.1245/s10434-024-16060-8

**Published:** 2024-08-17

**Authors:** Dirk Theile, David Czock

**Affiliations:** Internal Medicine IX, Department of Clinical Pharmacology and Pharmacoepidemiology, Heidelberg University Hospital, Heidelberg, Germany

With great interest, we read the recent article by Van der Speeten and co-workers reporting the results of an international consensus on technologies of hyperthermic intraperitoneal perioperative chemotherapy (HIPEC), published in *Annals of Surgical Oncology*.^1^ We compliment the authors for their endeavor, providing advice for HIPEC administration in clinical practice and also for clinical trials. Specifically, the target temperature (41–43°C), the dosimetry (mg/m^2^), the carrier solutions (e.g. saline for cisplatin), and perfusion times (e.g. 90 min) were agreed on. However, we would like to point out one potentially important aspect that may have been underrated: the perfusate flow rate (rarely reported, but often approximately 1–2 L/min.^2^). Our discussion will focus on cisplatin, but likely also applies to other drugs.

There are two main reasons why perfusate flow rate matters. First, the perfusate flow rate affects the heating of the perfusate, the peritoneum, and nearby tissues. With high flow rates, the perfusate is less efficiently heated up.^3^ However, once the target temperature is reached, high flow rates accelerate the heating of the peritoneum or esophagus.^4^ Despite its inconclusive clinical relevance, the effect of different flow rates on tissue temperature should be evaluated and discussed by the surgical community. Second, the perfusate flow rate likely affects cisplatin pharmacokinetics and thus the efficacy of the procedure. There seems to be an agreement that the efficacy of cisplatin HIPEC depends on the dose and duration of HIPEC, suggesting that the overall exposure of the peritoneal cancer tissue to the drug determines efficacy. Accordingly, high flow rate-mediated drug replenishment to the cancer cells in all parts of the abdomen should increase intracellular exposure, given the efficient drug transporter-mediated uptake of cisplatin into cancer cells.^5^ After its uptake, cisplatin is quickly transformed to positively charged moieties that readily react with nucleophilic targets such as DNA, constantly removing it from the cellular concentration equilibrium and further promoting its cellular uptake. This likely explains the good cisplatin penetration depth into peritoneal cancer tissue compared with other drugs,^1^ its good uptake into the systemic circulation (low peritoneal fluid/plasma area under the curve ratio^6^), and its known poor oral bioavailability.^7,8^ Together, cisplatin should be considered a high extraction drug. In accordance with high extraction drugs with high intrinsic hepatic clearance (e.g. by cytochrome P450 enzyme-mediated metabolism), flow rate should thus also matter for cisplatin during HIPEC administration. Moreover, tissues distant from the inflow sites could be exposed to low drug concentrations with low flow rates (because of inhomogeneous drug distribution in the abdomen), an effect that should be less with higher perfusate flow rates.

Physiology-based pharmacokinetic models previously simulated cisplatin kinetics during HIPEC; however, only two of six models eventually included the perfusate flow rate, which determines drug delivery to the peritoneal cavity. Unfortunately, these authors did not further elaborate on the relevance of the flow rate and its significance cannot conclusively be evaluated.^9^

In summary, there is good theoretical and experimental data suggesting that the perfusate flow rate might affect cisplatin uptake into the peritoneal lesions in different parts of the abdomen as well as into the systemic circulation. From a pharmacological point of view, the effects of different perfusate flow rates should also be evaluated, initially in animal models and also in clinical settings.
